# MR1-Independent Activation of Human Mucosal-Associated Invariant T Cells by Mycobacteria

**DOI:** 10.4049/jimmunol.1900674

**Published:** 2019-10-14

**Authors:** Sara Suliman, Melissa Murphy, Munyaradzi Musvosvi, Anele Gela, Erin W. Meermeier, Hennie Geldenhuys, Christiaan Hopley, Asma Toefy, Nicole Bilek, Ashley Veldsman, Willem A. Hanekom, John L. Johnson, W. Henry Boom, Gerlinde Obermoser, Huang Huang, Mark Hatherill, David M. Lewinsohn, Elisa Nemes, Thomas J. Scriba

**Affiliations:** * South African Tuberculosis Vaccine Initiative, University of Cape Town, Cape Town 7925, South Africa;; † Institute of Infectious Disease and Molecular Medicine, University of Cape Town, Cape Town 7925, South Africa;; ‡ Division of Immunology, Department of Pathology, University of Cape Town, Cape Town 7925, South Africa;; § Division of Rheumatology, Inflammation and Immunity, Brigham and Women’s Hospital, Harvard Medical School, Boston, MA 02115;; ¶ Department of Molecular Microbiology and Immunology, Oregon Health and Science University, Portland, OR 97239;; ‖ Tuberculosis Research Unit, Case Western Reserve University School of Medicine, Cleveland, OH 44106;; # Department of Medicine, Case Western Reserve University School of Medicine and University Hospitals Cleveland Medical Center, Cleveland, OH 44106;; ** Institute for Immunity, Transplantation and Infection, Stanford University School of Medicine, Stanford, CA 94305; and; †† Department of Microbiology and Immunology, Stanford University School of Medicine, Stanford, CA 94305

## Abstract

Tuberculosis (TB) is the leading cause of mortality from a single infectious agent, *Mycobacterium tuberculosis*. Relevant immune targets of the partially efficacious TB vaccine bacille Calmette–Guérin (BCG) remain poorly defined. Mucosal-associated invariant T (MAIT) cells are MHC-related protein 1 (MR1)–restricted T cells, which are reactive against *M. tuberculosis*, and underexplored as potential TB vaccine targets. We sought to determine whether BCG vaccination activated mycobacteria-specific MAIT cell responses in humans. We analyzed whole blood samples from *M. tuberculosis–*infected South African adults who were revaccinated with BCG after a six-month course of isoniazid preventative therapy. In vitro BCG stimulation potently induced IFN-γ expression by phenotypic (CD8^+^CD26^+^CD161^+^) MAIT cells, which constituted the majority (75%) of BCG-reactive IFN-γ–producing CD8^+^ T cells. BCG revaccination transiently expanded peripheral blood frequencies of BCG-reactive IFN-γ^+^ MAIT cells, which returned to baseline frequencies a year following vaccination. In another cohort of healthy adults who received BCG at birth, 53% of mycobacteria-reactive–activated CD8 T cells expressed CDR3α TCRs, previously reported as MAIT TCRs, expressing the canonical TRAV1-2-TRAJ33 MAIT TCRα rearrangement. CD26 and CD161 coexpression correlated with TRAV1-2^+^CD161^+^ phenotype more accurately in CD8^+^ than CD4^−^CD8^−^ MAIT cells. Interestingly, BCG-induced IFN-γ expression by MAIT cells in vitro was mediated by the innate cytokines IL-12 and IL-18 more than MR1-induced TCR signaling, suggesting TCR-independent activation. Collectively, the data suggest that activation of blood MAIT cells by innate inflammatory cytokines is a major mechanism of responsiveness to vaccination with whole cell vaccines against TB or in vitro stimulation with mycobacteria (*Clinical trial registration:* NCT01119521).

## Key Points

- MAIT cells comprise half the CD8 T cell IFN-γ response to BCG in blood.
- Frequencies of MAIT cells were not sustainably modulated by BCG vaccination.
- Innate cytokines mediate BCG-induced MAIT cell responses in whole blood.

## Introduction

According to the World Health Organization, tuberculosis (TB) is the leading cause of mortality from an infectious disease caused by a single pathogen, *Mycobacterium tuberculosis* (1). The only licensed and widely used vaccine against TB, bacille Calmette–Guérin (BCG), is given at birth in most TB-endemic countries and is partially efficacious against TB (2). It is not known which immune cell subsets or their features confer vaccine-mediated protection (3). Vaccine-induced Th1 CD4 T cells are routinely tested in clinical trials of candidate TB vaccines, but to date, such studies show that frequencies and functions of Th1 cells correlate poorly with vaccine efficacy (3). Relevant immune targets for vaccination remain poorly defined, particularly in *M. tuberculosis–*exposed individuals, which constitute almost one quarter of the world’s population (4).

Mucosal-associated invariant T (MAIT) cells are MHC-related protein 1 (MR1)–restricted innate T lymphocytes with antimicrobial properties (5, 6). MR1 presents riboflavin and folic acid metabolites derived from biosynthesis pathways in many bacteria, including mycobacteria and *Escherichia coli* (7). Most MAIT cells have a CD8^+^ or CD4^−^CD8^−^ phenotype (8, 9) and coexpress the CD26 peptidase (10) and C-type lectin CD161 (11, 12). MAIT cells predominantly express the invariant TCR α-chain TRAV1-2 (Vα7.2) (13) and a biased repertoire of TCR β-chains (14), although minor populations of TRAV1-2–negative MAIT cells have been reported (15, 16). MAIT cells can express IFN-γ, TNF-α, IL-17, and several cytotoxic effector molecules (17–19). MAIT cell clones were shown to respond to stimulation with *M. tuberculosis* in an MR1-dependent manner (20). Reduced frequencies of MAIT cells have been observed in the peripheral blood of active TB patients relative to healthy counterparts (5, 10, 21), and functional relevance for MAIT cells in controlling mycobacterial infection is supported by the finding that MR1-deficient mice have higher lung mycobacterial burden following aerosol challenge with *M. bovis* than MR1-sufficient counterparts (22). Interestingly, BCG vaccination of nonhuman primates transiently expanded frequencies of BCG-reactive MAIT cells in peripheral blood (23), suggesting that MAIT cells can be modulated by vaccination in a manner analogous to conventional HLA-restricted T cells. We previously reported that BCG vaccination at birth induced durable Ag-specific CD4 and CD8 T cell responses (24, 25). However, whether BCG-reactive T cells were HLA- or MR1-restricted and the implication of these restrictions on durability of vaccine-induced memory responses, remains unclear. MR1- and TCR-independent activation of MAIT cells via innate cytokines, such as IL-12, IL-18 (26, 27), and IFN-α (28), is well recognized. We previously showed that BCG revaccination of *M. tuberculosis*–infected adults induced long-term “memory” NK cell responses in such a cytokine-dependent manner (29). In this study, we sought to determine whether human MAIT cells respond to mycobacterial stimulation in vitro and to BCG vaccination in vivo. We also sought to determine whether these responses were mediated through MR1–TCR and/or cytokine-dependent mechanisms and to define the mechanisms underlying this response.

## Materials and Methods

### Human participants

#### BCG revaccination study.

We retrieved samples collected from tuberculin skin test (TST)-positive (with >15-mm induration at 48–72 h after PPD-RD23 injection), HIV-negative healthy South African adults who participated in a previously published phase I clinical trial (29, 30). Participants received a 6-mo course of isoniazid preventative treatment (West-Ward Pharmaceutical, Eatontown, NJ) prior to revaccination with BCG (Danish strain 1331 vaccination), delivered as an intradermal injection at the recommended dose for adults at 2–8 × 10^5^ CFUs. Heparinized whole blood was stimulated within 2 h of collection for the whole blood intracellular cytokine staining (WB-ICS) assay described below.

#### Adolescent cohort study.

We analyzed cryopreserved PBMC samples from a subset of participants of the previously reported Adolescent Cohort Study (31, 32). Healthy adolescents aged 12–18 y were recruited from high schools in Worcester, South Africa. PBMCs were collected using Sodium Heparin Vacutainer Cellular Preparation Tubes (BD Biosciences). Cells from participants with evidence of latent *M. tuberculosis* infection, determined by TST positivity (>15 mm induration) or QuantiFERON-TB Gold In-tube (≥0.35 IU/ml) were used to evaluate the concordance between frequencies of CD26^+^CD161^+^ MAIT cells and MR1 tetramer^+^ CD8 T cells as well as for single-cell sorting for TCR sequencing as described below.

#### Delayed BCG study.

We retrieved cryopreserved blood cells from 5- or 9-wk-old infants who received routine BCG vaccination at birth or in whom BCG vaccination was delayed until 6 or 10 wk of age, respectively. For the birth-vaccination group, mothers were approached at child vaccination clinics and asked to participate in the study. For the delayed BCG group, pregnant mothers were contacted antenatally and asked to participate in the study through hospitals in Worcester, South Africa. Infants of consenting mothers received an intradermal injection of the Danish strain 1331 of BCG at the standard infant dose of 1–4 × 10^5^ CFUs at either 6 or 10 wk. Heparinized blood was collected from infants in either group at 5 or 9 wk.

#### Healthy adult participants.

We recruited healthy adults over 18 y of age, who received BCG vaccination at birth. Heparinized blood was collected for WB-ICS assays to investigate TCR, MR1, and cytokine dependence of BCG-mediated MAIT cell activation.

### Ethics statement

All adult participants, parents or legal guardians of adolescents or infants, enrolled in the study provided written informed consent. Adolescents also provided written informed assent. The Medicines Control Council, now the South African Health products Regulatory Authority, or SAHPRA, of South Africa and the University Hospitals Cleveland Medical Center Institutional Review Board approved the phase I clinical trial of BCG revaccination, registered on ClinicalTrials.gov (NCT01119521). All remaining study protocols and blood collections were approved by the Human Research Ethics Committee of the University of Cape Town as follows: BCG revaccination trial (Ref. 387/2008), healthy infants and adults vaccinated at birth (Ref. 126/2006), infants with delayed BCG vaccination (Ref. 177/2011), and the Adolescent Cohort Study (Ref. 045/2005). We adhered to good clinical practice and the World Medical Association Declaration of Helsinki guidelines in the recruitment and treatment of all the study participants.

### WB-ICS assay

We processed heparinized whole blood for the standardized 12 h WB-ICS assay, as previously described (33, 34), within a maximum of 45 min from phlebotomy. Briefly, blood was stimulated with Ags at 37**°**C for 12 h. Brefeldin-A (10 μg/ml; Sigma-Aldrich, St. Louis, Mo.) was added for the final 5 h of stimulation. Stimulants included BCG Statens Serum Institut vaccine (1.2 × 10^6^ CFU/ml; The Biovac Institute, Cape Town, South Africa), DH5-α *E. coli* (dose titrated for maximal IFN-γ expression by MAIT cells), 15-mer peptide pools spanning ESAT-6/CFP-10 (1 μg/ml/peptide; Peptide Protein Research, London, United Kingdom). PHA was used as a positive control at 5 μg/ml, and RPMI 1640 only was the negative unstimulated control. Intracellular cytokine staining (ICS) assay stimulations were performed in the presence of anti-CD28 and anti-CD49d costimulatory Abs (each at 0.5 μg/ml; BD Biosciences). At the end of stimulation, blood was chelated with 20 μM EDTA (Sigma-Aldrich) and fixed with 1:10 FACS lysing solution (BD Biosciences) to lyse RBCs.

### In vitro cytokine stimulations and neutralization experiments

Cytokines used for whole blood stimulations included the following: recombinant human IL-2 (BD Biosciences), recombinant human IL-12p70 (eBioscience), recombinant human IL-18 (R&D Systems), and rIFN (eBioScience), each at a final concentration 0.1 μg/ml or lower, as determined by cytokine titration experiments. Neutralizing Abs included rat anti-human IL-2 (clone MQ1-17H12; BD Biosciences), mouse anti-human IL-12 (clone 24910; R&D Systems), mouse anti-human IL-18 (clone 125-2H; R&D Systems), LEAF anti-human/mouse/rat MR1 (catalog no. 361103; BioLegend, San Diego, CA), and mouse anti-human TCRα (clone T10B9.1A-31; BD Biosciences); the isotype controls included mouse IgG1 (clone 11711; R&D Systems) and rat IgG2a (clone 54447; R&D Systems). For type I IFN neutralization, we used vaccinia virus B18R Carrier-Free Recombinant Protein (eBioScience). All neutralizing Abs were used at a final concentration 10 μg/ml.

### Staining and acquisition by flow cytometry

Cryopreserved, fixed WBCs from adult or infant participants for the ICS assay were thawed and stained with one of the Ab panels in Perm/Wash buffer (BD Biosciences).

For PBMC samples, PE-conjugated MR1 tetramers loaded with 5-(2-oxopropylideneamino)-6-d-ribitylaminouracil (5-OP-RU) (35), obtained from the National Institutes of Health (NIH) tetramer core facility, were used to stain PBMC samples at 0.3 nM in 25 μl, as previously described (16), at room temperature for 45 min, followed by fluorochrome-conjugated Abs against surface markers at 4°C for 30 min. Samples were acquired on a BD LSR II flow cytometer configured with four lasers: Solid state Blue (488 nm; 100 mW; three detectors), Solid state Violet (405 nm; 25 mW; eight detectors), HeNe gas red (635 nm; 70 mW; three detectors), and diode-pumped Coherent Compass (532 nm; 150 mW; eight detectors). We used single-stained mouse κ-chain BD CompBeads to compensate all parameters. Individual Ab concentrations were titrated to volumes corresponding to their optimal stain index on the cytometer. The panels were further optimized in a Fluorescence Minus One analysis to maximize signal-to-noise ratios for each marker and minimize spectral overlap in the analyzed parameters. We acquired samples on optimal photomultiplier tube voltages calibrated daily using targets for SPHERO Rainbow Fluorescent Particles (Spherotech). All Ab mixtures are listed in Supplemental Table I.

### Single-cell TCR sequencing

Single-cell TCR sequencing was performed as described previously (36, 37). Briefly, cryopreserved PBMCs from 12 healthy adolescents with evidence of *M. tuberculosis* infection (determined by QuantiFERON-Plus and/or TST positive test results) were thawed, rested for 6 h, and stimulated for 12 h with *M. tuberculosis* lysate (10 μg/ml; BEI Resources) in the presence of anti-CD49d Ab (1 μg/ml), and anti-CD154-PE (10 μl/ml). Cells were stained with LIVE/DEAD Fixable Aqua Stain (Thermo Fisher Scientific) and then with Abs (Supplemental Table I). Single, activated (i.e., CD69^+^CD137^+^ and/or CD69^+^CD154^+^) TCRαβ^+^ CD8^+^ cells were sorted by FACS (BD FACSAria II) into 96-well plates containing OneStep RT-PCR buffer (QIAGEN). TCRαβ sequences were amplified using a panel of TCRαβ primers and further amplified in a nested PCR before sequencing on a MiSeq (Illumina) instrument, as described previously (36, 37). TCR sequences from six of these 12 adolescents were published recently (38), as indicated in Supplemental Table II.

### Analysis of public RNA-sequencing data from innate T cells

Public low-input RNA-sequencing data (Gene Expression Omnibus dataset: GSE124731) (39) from seven sorted cell populations (CD4^+^, CD8^+^, MAIT, invariant NK T cells, Vδ1^+^ and Vδ2^+^ γ∆ T cells, and NK cells) was mined using the online application in https://immunogenomics.io/itc/ to profile the expression of cytokine receptors on MAIT cells compared with the other immune subsets across the innate T cell gradient.

### Statistical analysis

We analyzed flow cytometry files using FlowJo versions 9.7–9.9 (Tree Star, Ashland, OR). Background subtractions were performed in Pestle version 1.7, and Boolean cytokine combinations were analyzed in SPICE version 5.3 (40). Statistical analyses and graphs were performed using Prism version 7 (GraphPad, La Jolla, CA) or R. We applied a Wilcoxon signed-rank test for paired analyses within the same participants and a Mann–Whitney *U* test for comparisons across different groups. Analysis and visualization of TCR pairs was performed using TCRdist (41). Assignment of TCR sequences to MAIT or conventional CD8 T cells was performed using the MAIT Match server (score = 1 for MAIT and <95% for conventional CD8 T cells), available at http://www.cbs.dtu.dk/services/MAIT_Match/ (accessed: October 30, 2018).

## Results

**FIGURE 1.**
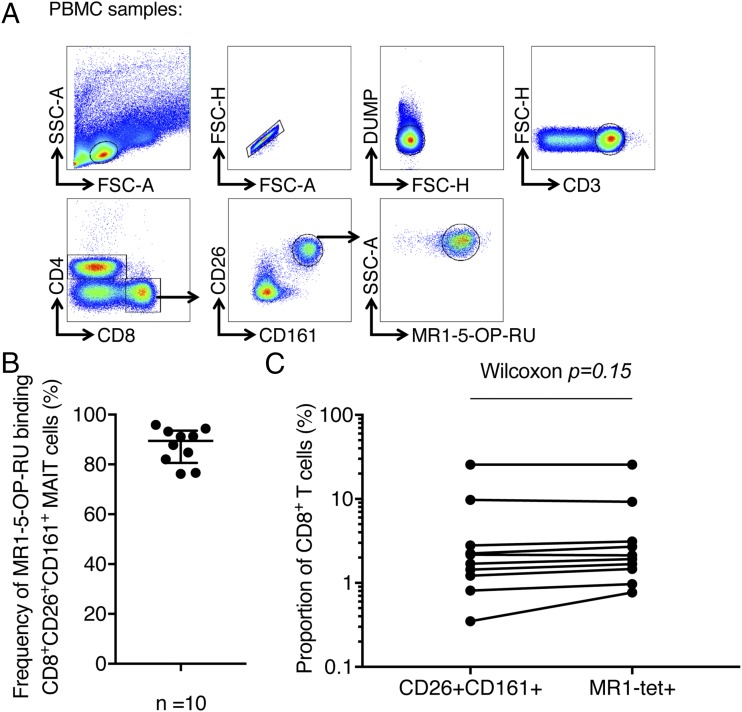
Identification of CD8^+^CD26^+^CD161^+^ MAIT cells and correlation with MR1 tetramer staining. (**A**) Gating strategy for PBMC used to estimate concordance of frequencies between 5-OP-RU–loaded MR1 tetramer staining and CD26^+^CD161^+^ phenotype as surrogate definitions for CD8^+^ MAIT cells. (**B**) Proportions of MR1–5OP-RU tetramer-binding CD8^+^ T cells, which stain positive for both CD26 and CD161 in PBMC samples from QuantiFERON-TB Gold (QFT)–positive South African adolescents (*n* = 10). The horizontal line depicts the median, and the error bars depict the ICR. (**C**) Paired frequencies of CD26^+^CD161^+^ and 5-OP-RU–loaded MR1 tetramer^+^ CD8 T cells in samples depicted in (B). The *p* values reflect a Wilcoxon signed-rank test between the paired frequencies from each donor.

We first established an appropriate in-house assay methodology to identify MAIT cells. MR1–5-OP-RU tetramer staining was suboptimal on samples processed for WB-ICS because leukocytes were fixed before cryopreservation. We therefore sought to use the phenotypic definition of CD8^+^ MAIT cells, based on coexpression of CD26 and CD161 (10, 11, 16), to identify CD8^+^CD26^+^CD161^+^ MAIT cells (Fig. 1A). A comparison of the two methods in healthy adult PBMC samples showed that the majority (median 89.5%, interquartile range [IQR] 82.7–92.7) of MR1­–5-OP-RU tetramer^+^ CD8^+^ T cells stained positive for CD26 and CD161 in adults and thus met the phenotypic definition (Fig. 1B). Frequencies of CD26^+^CD161^+^ cells and MR1 tetramer^+^ CD8^+^ T cells were not significantly different (Wilcoxon *p* *=* 0.15, Fig. 1C). Hence, we proceeded with the CD8^+^CD26^+^CD161^+^ phenotypic definition to demarcate CD8^+^ MAIT cells in fixed whole blood samples for subsequent ICS analyses.

**FIGURE 2.**
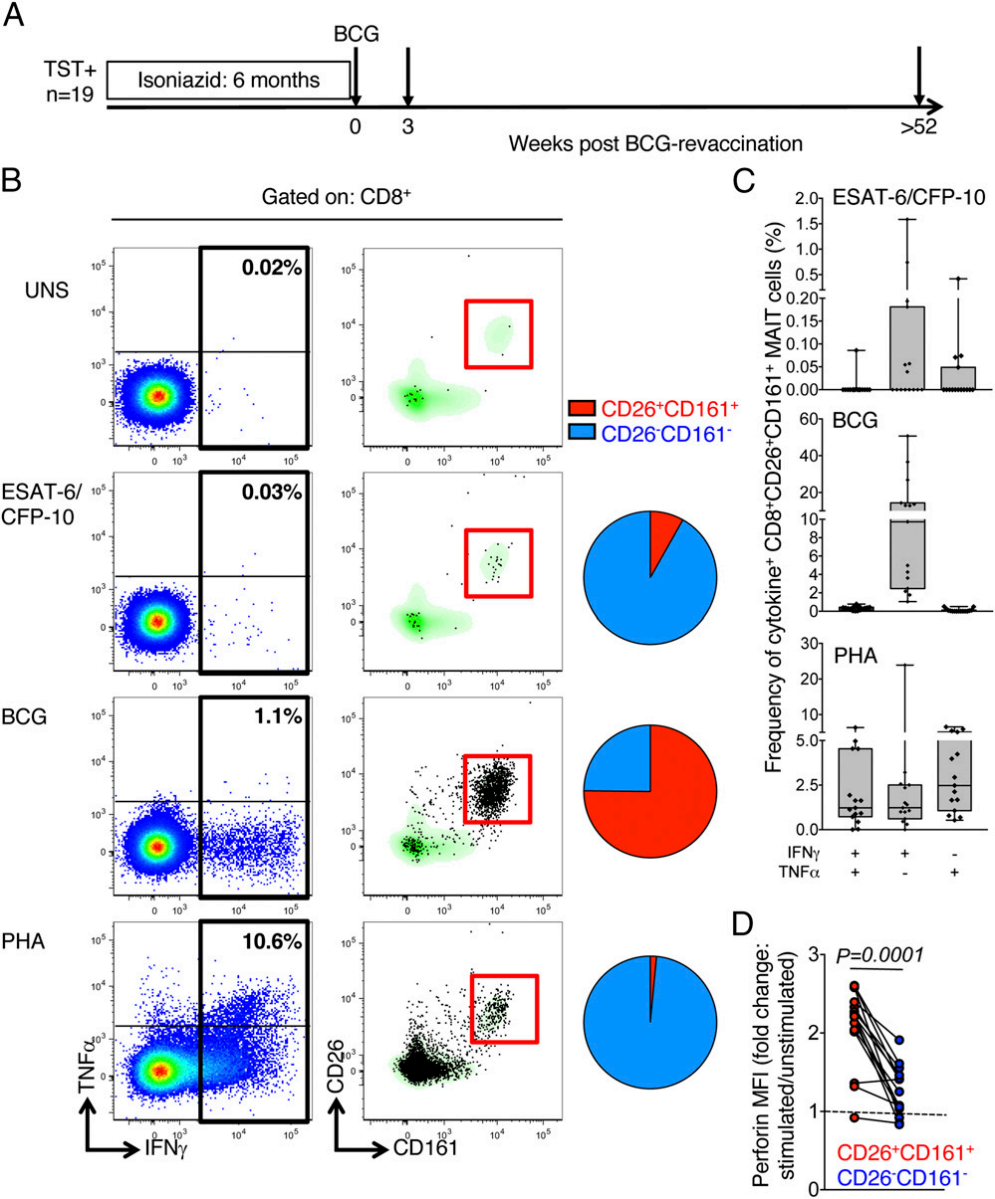
CD8^+^ MAIT cell responses to in vitro BCG stimulation. (**A**) Study design for BCG revaccination: individuals with TST induration >15 mm were revaccinated with BCG after 6 mo of isoniazid treatment. Samples for this study were analyzed prevaccination (0 wk), 3, and 52 wk postvaccination (denoted by arrows). (**B**) Representative flow cytometry plots of CD8 T cells measured by WB-ICS after stimulation with the conditions indicated on the left (from top to bottom: UNS [unstimulated as negative control], ESAT-6/CFP-10 peptide pools, BCG, and PHA as positive control). Plots on the left are gated on CD3^+^CD8^+^ T lymphocytes. Plots on the right overlay IFN-γ^+^ cells (black dots), as gated in the thick black boxes on the left, over total CD3^+^CD8^+^ cells (green density plots). The pie charts correspond to the mean frequencies of IFN-γ^+^ CD26^+^CD161^+^ (red) or CD26^−^CD161^−^ (blue) CD8 T cells for each stimulation condition. (**C**) Frequencies of CD8^+^CD26^+^CD161^+^ cells coexpressing different combinations of IFN-γ and/or TNF-α. Frequencies in unstimulated samples were subtracted from their corresponding Ag-stimulated samples. Horizontal lines represent the median, the boxes represent the IQR, and the whiskers represent the range. (**D**) Per cell perforin expression in either CD26^+^CD161^+^ (red) or CD26^−^CD161^−^ (blue) CD8^+^ T cells expressed as median fluorescent intensity fold change in BCG-stimulated cells over unstimulated cells. The *p* values correspond to a Wilcoxon signed-rank test of paired values within each sample.

Several studies of BCG vaccination have reported the presence of BCG-reactive cytokine-expressing CD8 T cells in peripheral blood, which were largely interpreted to be MHC class I–restricted T cells (24, 25, 42). Because MAIT cells comprise 5–10% of circulating CD8^+^ T cells (13), we sought to identify the relative contribution of MAIT cells to cytokine-expressing BCG-reactive CD8 T cells in *M. tuberculosis*–sensitized healthy adult participants of the BCG revaccination trial (Fig. 2A), (29, 30). Following ESAT-6 and CFP-10 peptide pool stimulation, CD26^−^CD161^−^ CD8 T cells comprised the majority (median 91.8%, IQR 85.2–98.3) of the total IFN-γ–expressing CD8 T cell population in samples collected before BCG revaccination (Fig. 2B). Unexpectedly, however, following BCG stimulation, most IFN-γ–expressing CD8 T cells were CD26^+^CD161^+^ MAIT cells (median 75.2%, IQR 55.8–84.4, Fig. 2B). By comparison, PHA-stimulated IFN-γ–expressing CD8 T cells were almost entirely among the CD26^−^CD161^−^ CD8 T cell fraction (median 98.5%, IQR 98–99.3). Further, BCG-reactive IFN-γ–positive CD8^+^CD26^+^CD161^+^ MAIT cells coexpressed very little TNF-α (Fig. 2C). Because MAIT cells typically have high cytotoxic potential (18), we also compared perforin expression. Perforin expression after BCG stimulation increased to a significantly higher degree in CD26^+^CD161^+^ CD8^+^ MAIT cells than in their CD26^−^CD161^−^ CD8 T cell counterparts (Fig. 2D). These data collectively support that CD26^+^CD161^+^ CD8^+^ MAIT cells in *M. tuberculosis*–sensitized adults respond to BCG by expressing IFN-γ and perforin.

**FIGURE 3.**
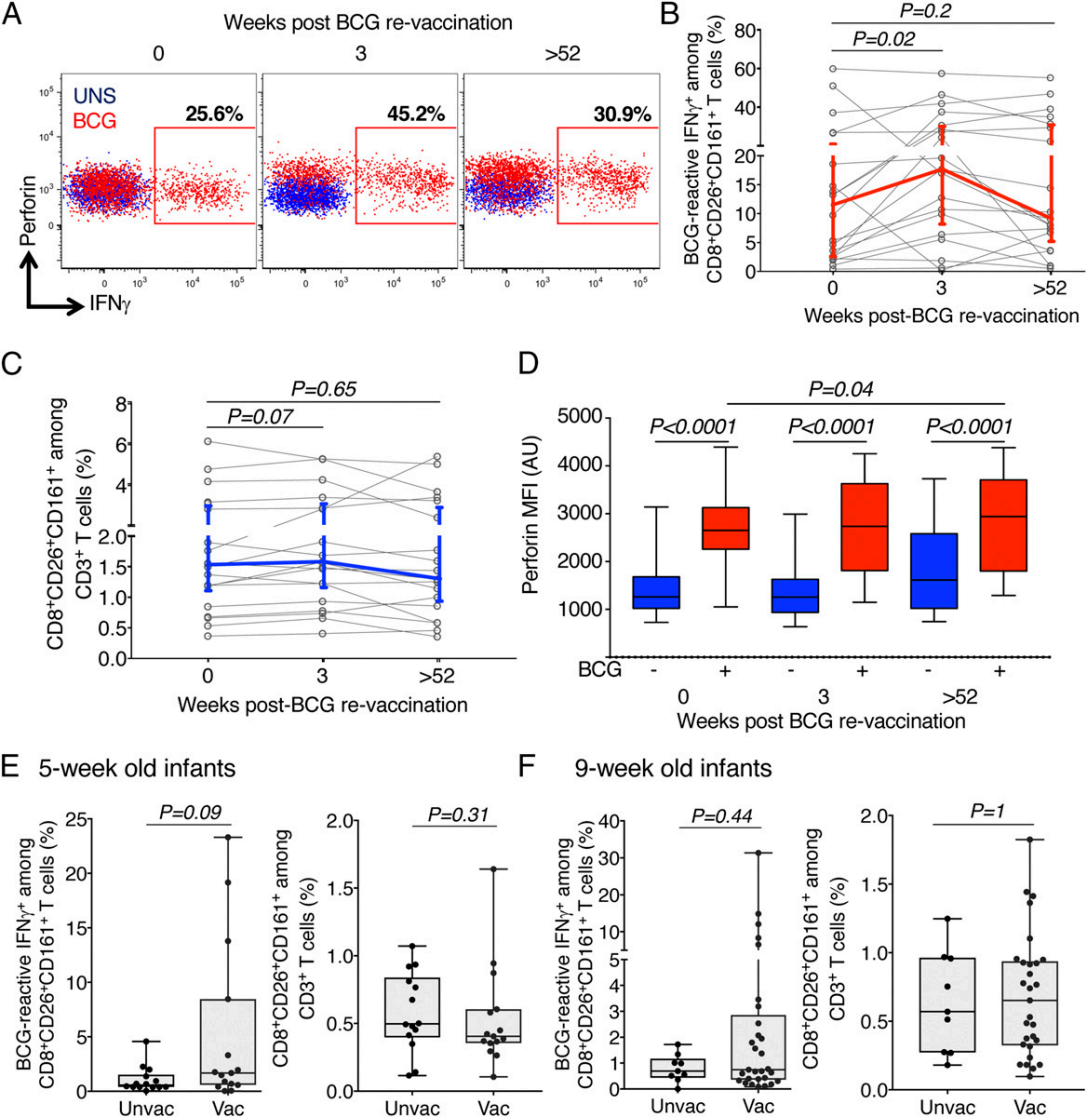
Changes in MAIT cell function after BCG revaccination. (**A**) Representative flow cytometry plots showing expression of IFN-γ and perforin in CD26^+^CD161^+^ CD8^+^ T cells from BCG-revaccinated adults. Red boxes demarcate IFN-γ–positive cells in BCG-stimulated whole blood (red dots) overlaid onto unstimulated blood (blue dots). Plots correspond to samples taken prevaccination (left), 3 wk (middle), and 1 y (right) following BCG revaccination. (**B**) Frequencies of BCG-reactive IFN-γ–expressing CD26^+^CD161^+^ CD8^+^ T cells in individual study participants before (week 0) and after vaccination (weeks 3 and >52). Red line and error bars show median frequencies and IQR in the cohort, respectively. The *p* values were calculated using a Wilcoxon signed-rank test for paired values. (**C**) Frequencies of CD26^+^CD161^+^ CD8^+^ T cells (as a proportion of CD3^+^ T cells) in individual study participants before (week 0) and after vaccination (weeks 3 and >52). Blue line and error bars denote the median and IQR, respectively. The *p* values were calculated using a Wilcoxon signed-rank test for paired values. (**D**) Median fluorescence intensities of perforin expression in CD26^+^CD161^+^ CD8^+^ T cells in unstimulated (blue) or BCG-stimulated (red) whole blood samples at prevaccination, 3 wk and 1 y following BCG revaccination. *p* values were calculated using Wilcoxon signed-rank test for paired unstimulated and BCG-stimulated samples at each timepoint and between BCG-stimulated samples prevaccination and 1 y after vaccination. Horizontal lines represent the median, the boxes represent the IQR, and the whiskers represent the range. (**E** and **F**) Frequencies of BCG-reactive IFN-γ–expressing CD8^+^CD26^+^CD161^+^ T cells (left panel) or proportions of CD8^+^CD26^+^CD161^+^ in total T cells (right panel) in 5-wk-old (E) or 9-wk-old (F) infants who were BCG-naive at the time of sample collection (Unvac) or BCG-vaccinated at birth (Vac).

Because MAIT cells respond robustly to BCG stimulation in vitro and possess effector functions that can kill or control intracellular *M. tuberculosis* (18), they pose a potentially attractive candidate target for vaccination against TB. Hence, we evaluated whether BCG vaccination would expand frequencies of BCG-reactive MAIT cells in a manner previously reported for conventional memory T cells (24, 29). As a proof of principle, we evaluated whether frequencies of BCG-reactive CD26^+^CD161^+^ CD8^+^ MAIT cells could be boosted in vivo by BCG revaccination of *M. tuberculosis*–sensitized adults (Fig. 2A). Interdonor frequencies of BCG-reactive MAIT cells expressing IFN-γ were highly heterogeneous, ranging from 0 to 60% of the total CD8 T cell subset (Fig. 3A, 3B). These IFN-γ–expressing BCG-reactive MAIT cells were transiently boosted 3 wk following BCG revaccination, thereafter retracting to prevaccination frequencies (Wilcoxon *p* *=* 0.2, Fig. 3A, 3B). We observed the same trend when frequencies of IFN-γ^+^ CD8^+^CD26^+^CD161^+^ MAIT cells for each individual were normalized to their prevaccination frequencies (data not shown). This transient increase in frequencies of IFN-γ–expressing BCG-reactive MAIT cells was not associated with expansion of CD8^+^CD26^+^CD161^+^ MAIT cell proportions within CD3^+^ T cells, suggesting that vaccination modulated IFN-γ expression independently of the peripheral blood proportions of CD8^+^CD26^+^CD161^+^ MAIT cells (Fig. 3C). BCG stimulation in vitro also significantly increased per cell levels of perforin expression by CD8^+^CD26^+^CD161^+^ MAIT cells at any given timepoint in the trial (Fig. 3D). Interestingly, these levels of perforin expression by CD8^+^CD26^+^CD161^+^ MAIT cells were modestly higher in samples collected 1 y after BCG revaccination than in prevaccination samples (Fig. 3D).

It is possible that mycobacteria-reactive CD8^+^CD26^+^CD161^+^ MAIT cells were already pre-expanded in *M. tuberculosis*–sensitized adults because of the underlying bacterial infection, thereby masking any additional reactivity or expansion conferred by BCG vaccination. Therefore, we tested whether BCG-reactive CD8^+^CD26^+^CD161^+^ MAIT cells could be expanded by BCG vaccination in infants without prior exposure to mycobacteria. We analyzed BCG-stimulated whole blood samples collected from 5-wk-old infants who received BCG at birth or at 6 wk of age and were, therefore, naive to mycobacteria at the time of sample collection (Fig. 3E). To maintain a consistent phenotypic definition of MAIT cells, we only analyzed CD8^+^CD26^+^CD161^+^ cells. Frequencies of BCG-reactive IFN-γ–expressing CD8^+^CD26^+^CD161^+^ cells in unvaccinated and vaccinated infants were not statistically different (Wilcoxon *p* = 0.09; Fig. 3E, left). Similar to adults, proportions of T cells that had a CD8^+^CD26^+^CD161^+^ phenotype were similar between the two groups (Wilcoxon *p* = 0.31; Fig. 3E, right), suggesting that BCG did not mediate a relative expansion of CD8^+^CD26^+^CD161^+^ among T cells. We observed the same result in an independent cohort of 9-wk-old infants (Fig. 3F).

**FIGURE 4.**
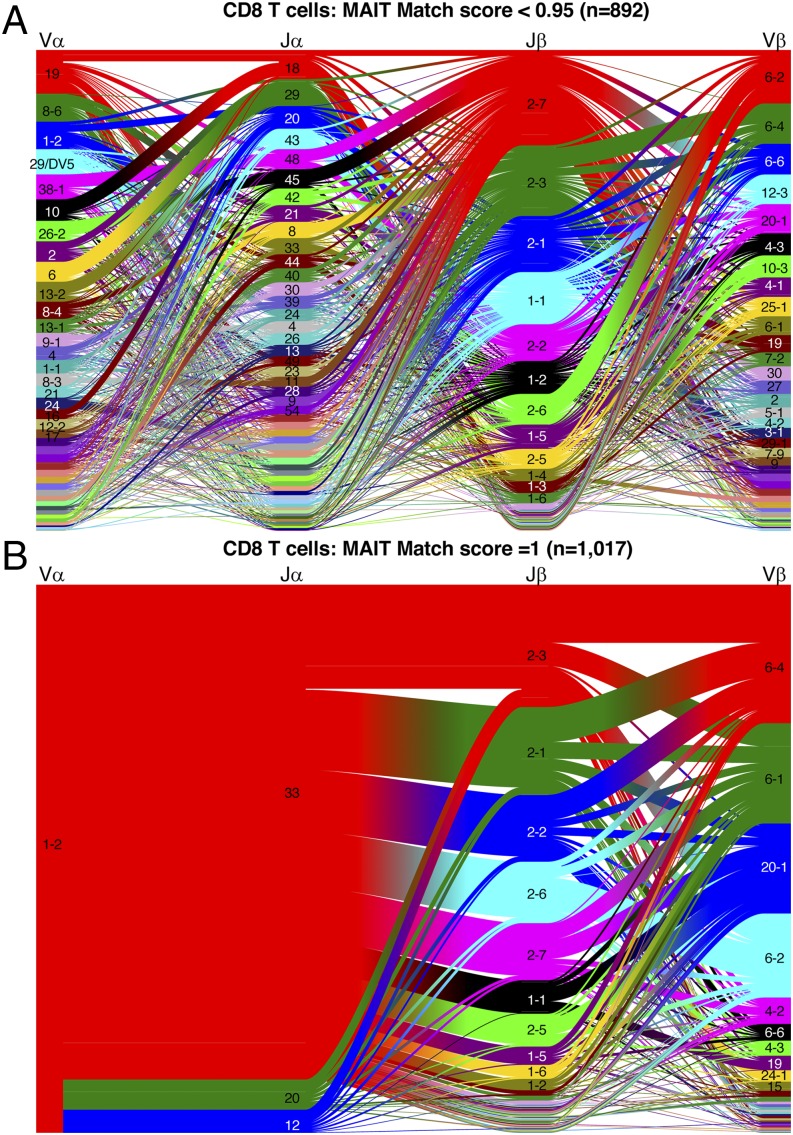
Mycobacteria-reactive MAIT CD8 T cells express canonical MAIT TCR sequences. Variable (V) and joining (J) gene segment usage for TCR α and β pairs in single *M. tuberculosis* lysate-activated (CD69^+^CD154^+^ or CD69^+^CD137^+^) CD8 T cells that (**A**) do not conform to canonical MAIT CDR3α sequences (MAIT Match score <0.95; *n* = 892) or (**B**) show an exact match to known MAIT CDR3α clonotypes (MAIT Match score = 1; bottom, *n* = 1017). Gene segment usage and gene–gene pairing landscapes are illustrated using four vertical stacks (one for each V and J segment) connected by curved paths in which thickness is proportional to the number of TCR clones with the respective gene pairing. Genes are colored by their relative proportion among sorted single cells. Red (most frequent), green (second-most frequent), blue, cyan, magenta, and black, etc.

To delineate the identity of mycobacteria-reactive CD8^+^ T cells further, we sequenced the TCRs of single CD8 T cells that were activated by in vitro stimulation with *M. tuberculosis* lysate. CD137 and CD154 were reported to mark activated conventional and innate-like CD8 T cells, respectively (38). CD69^+^CD137^+^ and/or CD69^+^CD154^+^ CD8 T cells were sorted, and the TCRα and TCRβ loci were sequenced as previously described (37) (Supplemental Fig. 1A, Supplemental Table I). A total of 1909 single CD8^+^ T cells with paired TCRα and TCRβ sequences from 12 healthy latently *M. tuberculosis*–infected adolescents were identified (Supplemental Table II), including public TCR sequences from six donors (38). CDR3α sequences were analyzed using the “MAIT Match” algorithm (43), which calculates a similarity score relative to known MAIT cell TCR sequences (http://www.cbs.dtu.dk/services/MAIT_Match/). CD8^+^ T cell clones with CDR3α sequences that had a MAIT Match score of <0.95 or one were classified as conventional (Fig. 4A), or CD8 MAIT cells (Fig. 4B), respectively. Interestingly, 53% (1017/1909) of TCR sequences derived from CD8 T cells activated by stimulation with *M. tuberculosis* lysate expressed previously published, canonical MAIT CDR3α TCR sequences, all of which expressed the canonical TRAV1-2 variable chain, and most expressed the TRAJ33 segment, with a few expressing TRAJ12 or TRAJ20, consistently with reported MAIT TCRα rearrangements (11, 14, 44, 45). Although the TCRβ gene usage by these CD8 MAIT cells was more diverse, a biased repertoire consistent with reported MAIT TCRβ sequences was also observed (14, 46). By comparison, activated CD8 T cells with CDR3α MAIT Match scores of <0.95 displayed markedly more diverse VαJα gene usage, consistent with conventional, MHC class I–restricted CD8 T cells (Fig. 4A). These data confirm that MAIT CD8 T cells with the canonical TCR α-chain are activated by stimulation with a mycobacterial Ag preparation.

**FIGURE 5.**
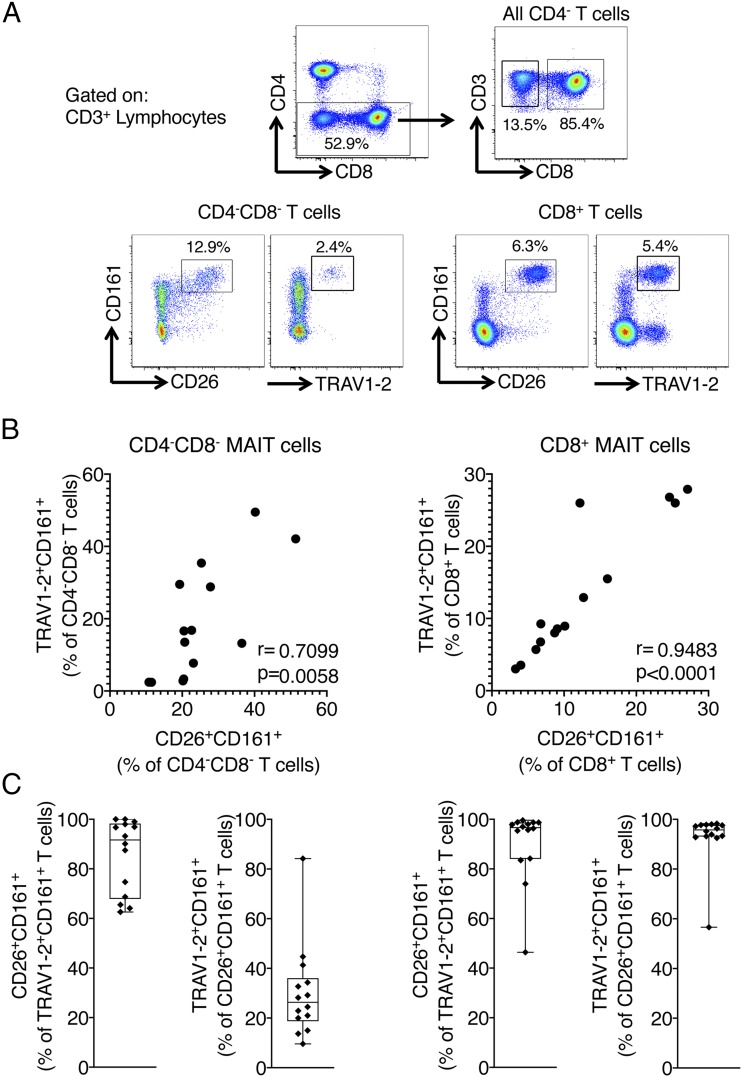
Concordance between CD26^+^CD161^+^ and TRAV1-2^+^CD161^+^ phenotypes in CD4^−^CD8^−^ and CD8^+^ MAIT cells. (**A**) Flow cytometry gating strategy to identify CD4^−^CD8^−^ (bottom left) and CD8^+^ (bottom right) MAIT cells from total CD4^−^ T lymphocytes. MAIT cells are gated using either CD26**^+^**CD161**^+^** and TRAV1-2**^+^**CD161**^+^** phenotypic definitions. (**B**) Correlation between frequencies of CD26**^+^**CD161**^+^** and TRAV1-2**^+^**CD161**^+^** cells among total CD4^−^CD8^−^ (left) or CD8**^+^** T cells (right). The *p* values and correlation coefficients are calculated using the nonparametric Spearman correlation test. (**C**) Box and whisker plots showing proportions of CD26**^+^**CD161**^+^** cells among TRAV1-2**^+^**CD161**^+^** cells and converse proportions of TRAV1-2**^+^**CD161**^+^** among CD26**^+^**CD161**^+^** cells within CD4^−^CD8^−^ (left) or CD8**^+^** T cells (right). The horizonal lines, boxes, and error bars correspond to median proportions, IQR, and range, respectively.

Previous studies characterizing MAIT cells in TB defined MAIT cells as CD161^+^TRAV1-2^+^ (5, 21). Further, MAIT cells include both CD8^+^ and CD4^−^CD8^−^ subsets (8), which were recently shown to be functionally distinct (9). Because we did not include anti–TRAV1-2 and anti-CD4 Abs to stain MAIT cells in the clinical trial of BCG revaccination (Figs. 2, 3), we recruited healthy BCG-vaccinated adult donors to validate our CD8^+^CD26^+^CD161^+^ definition of MAIT cells and compare the responses of CD4^−^CD8^−^ and CD8^+^ MAIT cells to BCG (Fig. 5A, Supplemental Fig. 1B). Frequencies of CD8^+^ CD26^+^CD161^+^ and CD8^+^ TRAV1-2^+^CD161^+^ subsets were very strongly correlated in T cells (Spearman *r* = 0.95), but among CD4^−^CD8^−^ T cells, these subsets were less strongly correlated (Spearman *r* = 0.71, Fig. 5B). The CD26^+^CD161^+^ phenotype thus accurately captured TRAV1-2^+^CD161^+^ MAIT cells in CD8^+^ but not CD4^−^CD8^−^ T cells (Fig. 5C).

**FIGURE 6.**
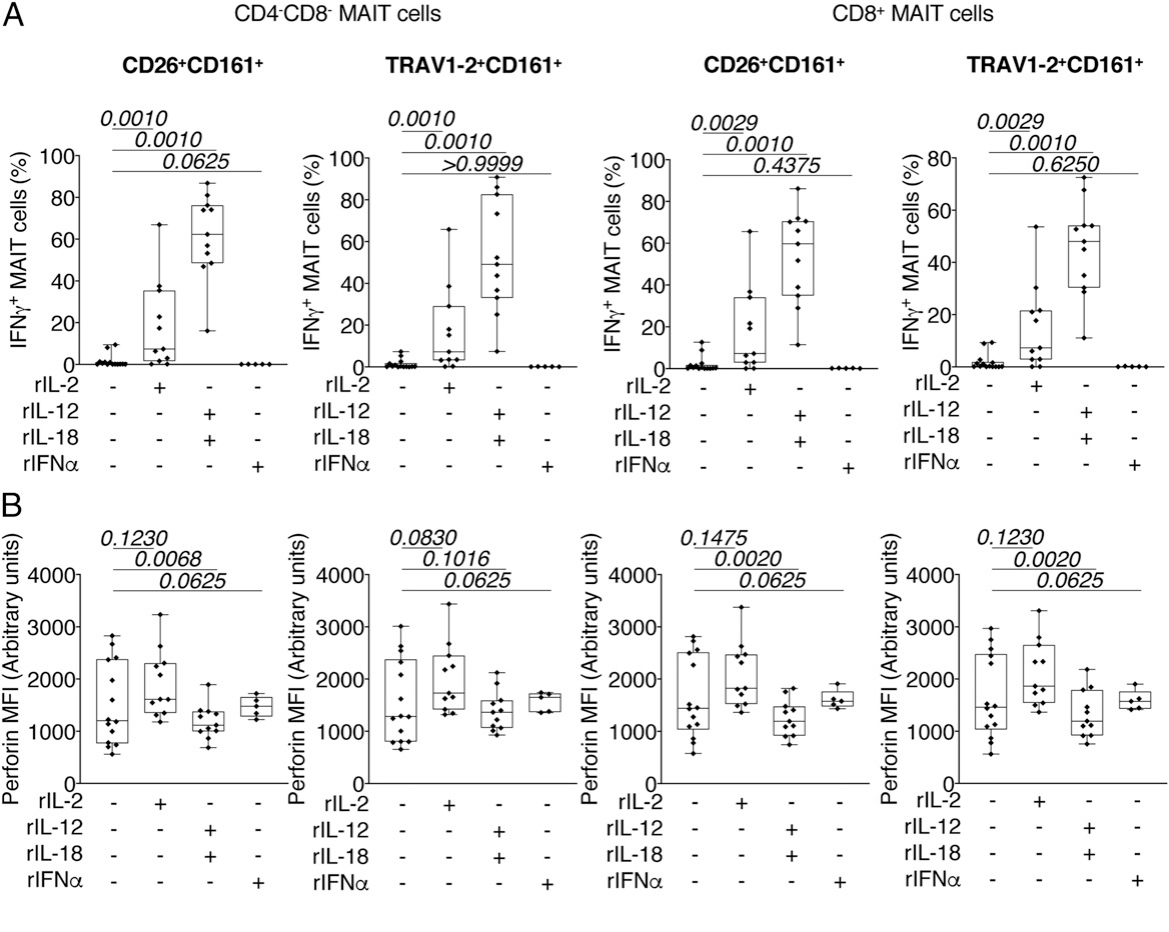
Blood MAIT cell activation in response to stimulation with cytokines. (**A**) Frequencies of IFN-γ–positive CD4^−^CD8^−^ or CD8**^+^** T MAIT subsets identified either as CD26**^+^**CD161**^+^** or TRAV1-2**^+^**CD161**^+^** cells in unstimulated control samples or in response to stimulation with 100 ng of recombinant human IL-2, a combination of IL-12 and IL-18, or rIFN-α. (**B**) Median fluorescence intensities corresponding to perforin expression under the same cytokine stimulation conditions and MAIT subset definitions used in (A). For both (A) and (B), *p* values were calculated using the Wilcoxon signed-rank test. Horizontal lines represent the median, the boxes represent the IQR, and the whiskers represent the range.

MAIT cells can be activated by stimulation with innate cytokines, including IL-12 and IL-18 (26, 27), or type I IFN (IFN-α) (28). Published RNA-sequencing data (39) demonstrate that MAIT cells express high levels of the IL-12Rβ and IL-18R1 cytokine receptors relative to other T cell subsets (Supplemental Fig. 2A) and are thus likely to respond to innate inflammatory cytokines induced by BCG stimulation (47). We tested the responses of CD4^−^CD8^−^ and CD8^+^ MAIT cells in whole blood to stimulation with these cytokines in vitro (Fig. 6). Stimulation with recombinant human IL-12 and human IL-18, or human IL-2 alone to a lesser extent, but not rIFN-α, significantly increased IFN-γ expression in both CD4^−^CD8^−^ and CD8^+^ MAIT cells, whether they were defined as CD26^+^CD161^+^ or TRAV1-2^+^CD161^+^ cells (Fig. 6A). IFN-γ expression in response to IL-12 and IL-18 stimulation was dose dependent (Supplemental Fig. 2B, 2C) and synergistic (Supplemental Fig. 2D). Unexpectedly, however, stimulation with IL-12 and IL-18 reduced perforin expression in an Ag-independent manner, especially in CD8^+^ MAIT cells (Fig. 6B).

**FIGURE 7.**
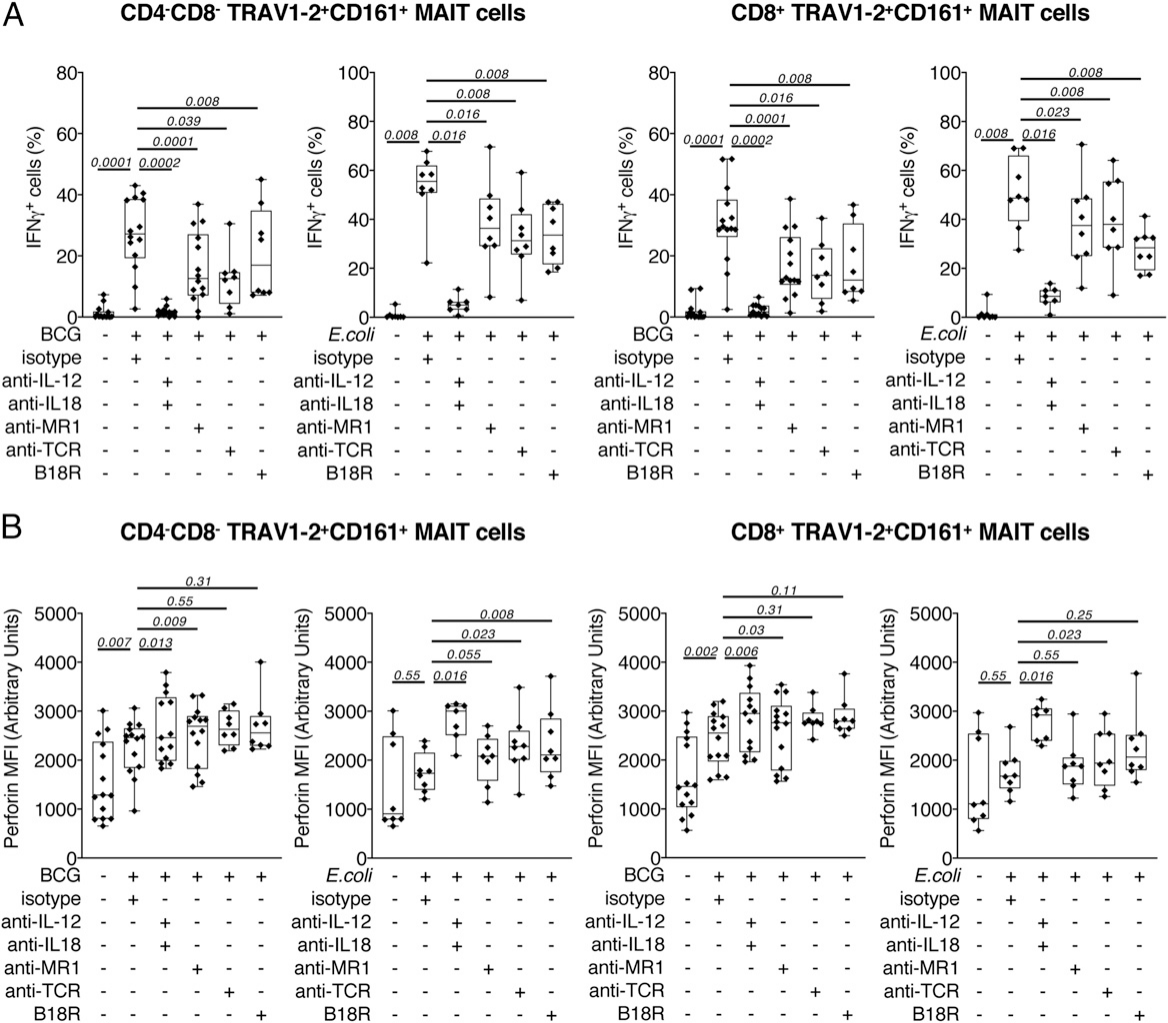
Dependence of BCG or *E. coli*–induced MAIT cell activation on innate cytokines. (**A**) Box and whisker plots showing frequencies of IFN-γ–positive CD4^−^CD8^−^ or CD8**^+^** T cell MAIT subsets, all identified in this study as TRAV1-2**^+^**CD161**^+^** cells, in response to stimulation with BCG or *E. coli* in the presence of isotype control Abs or neutralizing Abs against IL-12 and IL-18, MR1, TCR, or the type 1 IFN antagonist (B18R). (**B**) Median fluorescence intensities corresponding to perforin expression under the same stimulation conditions as (A). For both (A) and (B), *p* values were calculated using the Wilcoxon signed-rank test.

Next, we examined whether in vitro MAIT cell responses to mycobacterial stimulation in whole blood were dependent on signaling through the MR1–TCR interaction or inflammatory cytokines previously reported to be induced by BCG (48, 49). We analyzed the requirement of these signals for MAIT cell activation in whole blood downstream of stimulation with BCG or *E. coli*, which is known to activate MAIT cells (50) (Fig. 7). We focused on TRAV1-2^+^CD161^+^ cells because this definition identified MAIT cells more comprehensively among CD4^−^CD8^−^ T cells (Fig. 5B, 5C). Stimulation of blood with BCG or *E. coli* in the presence of anti–IL-12 and anti–IL-18 Abs completely abrogated IFN-γ expression in either CD4^−^CD8^−^ or CD8^+^ MAIT cells (Fig. 7A). Neutralization of MR1 or TCR signals only partially blocked BCG or *E. coli*–induced IFN-γ expression, even when increasing the concentration of anti-MR1 blocking Ab 4-fold relative to published methods (Supplemental Fig. 2E). Blocking IFN-α with the recombinant B18R protein, a vaccinia virus-encoded decoy type I IFNR (51) (Fig. 7A) or IL-2 neutralization (Supplemental Fig. 2F) also only partially reduced frequencies of IFN-γ expression in CD4^−^CD8^−^ or CD8^+^ MAIT cells in Ag-stimulated blood samples. Consistent with our IL-12 and IL-18 stimulation experiment (Fig. 6B), blocking IL-12 and IL-18 significantly upregulated perforin expression in CD4^−^CD8^−^ or CD8^+^ MAIT cells from BCG or *E. coli*–stimulated whole blood samples (Fig. 7B).

Collectively, our data suggest that upon in vitro bacterial stimulation of whole blood IL-12 and IL-18 cooperatively mediated more potent MAIT cell IFN-γ expression compared with either TCR/MR1–mediated signals or other cytokines known to induce MAIT cell activation.

## Discussion

MR1-restricted MAIT cells have recently gained considerable attention for their diverse range of antimycobacterial functions (52). Understanding mechanisms underlying activation of MAIT cells would inform whether these cells should be targeted by vaccination and shed new insights into rational vaccine design for a complex human disease such as TB. Our study highlights three points of relevance to T cell responses to mycobacteria with implications for vaccination strategies against TB. First, we showed that MAIT cells comprised the majority of mycobacteria-reactive CD8 T cells in the peripheral blood of *M. tuberculosis*–sensitized individuals. Second, BCG vaccination of either adults or infants from a TB-endemic region did not induce durable changes in BCG-reactive MAIT cell frequencies in a manner consistent with T cell memory. Finally, BCG-induced activation of MAIT cells was mediated predominantly by the innate cytokines IL-12 and IL-18 in stimulated blood in vitro and to a lesser degree from other signals, including MR1–TCR triggering, IL-2, and type I IFN, suggesting bystander activation of MAIT cells as a major mechanism for IFN-γ production in response to mycobacteria.

Historically, studies of BCG-reactive T cells following vaccination have not aimed to distinguish between classical HLA-restricted and other unconventional populations of T cells, such as MR1-restricted MAIT cells (24, 25, 42). We defined MAIT cells phenotypically as CD8^+^CD26^+^CD161^+^ in the first part of this study, which captured most CD8^+^ T cells that bound MR1 tetramers and are strongly correlated with TRAV1-2 and CD161 coexpression. This concordance is consistent with the reported high accuracy of CD26 and CD161 coexpression to identify CD8^+^ MR1 tetramer-binding MAIT cells but not CD4^−^CD8^−^ counterparts (11). Our findings show that historical analyses of bulk CD8 T cell responses activated by mycobacterial stimulation or vaccination overlooked the relative contribution of classical MHC-I–restricted and MR1-restricted CD8 T cells and thus eclipsed the contribution of MAIT cells to the overall antimycobacterial response by CD8 T cells (24, 29, 42, 53). This finding has important implications for understanding the mechanism underlying BCG-mediated host responses. Although we did not observe durable increases in frequencies of BCG-reactive MAIT cells, bystander activation of MAIT cells and, potentially, other innate lymphocytes, as we previously demonstrated for NK cells (29), suggest that mechanisms other than clonal expansion of peptide-specific memory T cells could play a role in the antimycobacterial immune response and should be considered in rational TB vaccine design (54).

Interestingly, our data suggest that both newborn BCG vaccination of naive infants as wells as BCG revaccination of *M. tuberculosis*–infected adults did not induce durable changes in frequencies of mycobacteria-reactive IFN-γ–expressing MAIT cells. A similar finding was observed in BCG-vaccinated nonhuman primates, which only transiently expanded MR1 tetramer-positive cells after vaccination (23). We cannot rule out that vaccination may have expanded a specific subset of MAIT cell clones that we could not discern by measuring the overall size of BCG-reactive MAIT cells. For example, a recent study of a controlled *Salmonella enterica serovar Paratyphi A* infection of human volunteers showed that the population size of MR1-restricted T cells remained consistent postinfection, although certain MAIT TCR sequences were expanded in individuals who developed disease (46). In our adult study, mycobacteria-reactive MAIT cells may have already expanded after initial exposure to *M. tuberculosis*, especially because participants showed high-level immune sensitization to *M. tuberculosis* (29). Hence, clonal expansion of MAIT TCR sequences following BCG vaccination would be better analyzed in a cohort of mycobacteria-naive donors from a non–TB-endemic setting and low exposure to environmental mycobacteria so as not to mask BCG-specific clonal expansion of MAIT cells (55). However, the poor induction of IFN-γ expression by BCG-stimulated CD8^+^CD26^+^CD161^+^ in BCG-vaccinated infants relative to mycobacteria-naive counterparts argues against this possibility and suggests that BCG does not induce or is poor at inducing memory MAIT cell responses.

*E. coli* has an intact riboflavin biosynthesis pathway and can thus generate relevant MR1 ligands to activate MAIT cell TCRs (7, 35). A recent study characterized ligands eluted from MR1 monomers generated in cells infected with *E. coli* or *M. smegmatis* and identified a broader range of putative MR1 ligands than previously appreciated (56). BCG or *E. coli*–derived MR1 Ags could theoretically activate MAIT cells via MR1–TCR triggering. However, follow-up studies identifying and knocking out the genetic analogs of riboflavin biosynthetic enzymes (35) in *M. bovis* and *M. tuberculosis* are essential to formally validate that mycobacteria can directly generate MR1 Ags because the enzymes have only been inferred to be intact in these mycobacterial species based on Kyoto Encyclopedia of Genes and Genomes annotations (57). The partial blocking of MAIT cell activation by MR1 and TCR neutralization argues against our Ag preparations not containing sufficient concentrations of MR1 ligands required for MR1–TCR triggering. However, we show that stimulation of whole blood samples with either organism induced IFN-γ expression by MAIT cells that was predominantly mediated by IL-12 and IL-18 signals. Although we employed an in vitro assay to characterize functional MAIT cell responses, whole blood provides a physiologically relevant approximation of in vivo responses (26, 27, 58). Additionally, IL-12 is produced by monocytes in BCG-stimulated whole blood samples of vaccinated infants (47, 59), suggesting that bystander MAIT cell responses to BCG could be physiologically relevant. MAIT cells have innate-like transcriptional programming (9, 12), and have been shown to express IFN-γ downstream of these innate cytokines (26, 39). Furthermore, a prior report suggested that viral infection activated MAIT cells indirectly via IL-18 synergistically with type I IFN signals (28). We found that type I IFN–mediated signals only partially activated MAIT cells after mycobacterial stimulation. Modulation of perforin expression in response to IL-12 and IL-18 followed the opposite trend to IFN-γ, which we propose to indicate T cell degranulation. Collectively, MAIT cells are poised to preferentially respond to innate inflammatory signals, consistent with their reported transcriptional program (12, 39). Because MAIT cells are enriched in airways (60), they could act as sentinels for initial infection by responding to danger signals from infected macrophages and dendritic cells, mediating activation of *M. tuberculosis*–infected macrophages through cytokine expression. MAIT cells would then initiate recruitment of other immune cells, including Ag-specific CD4 T cells, although this association was variable in *M. tuberculosis*–infected macaques (61). Given that *M. tuberculosis* is known to subvert peptide Ag presentation by macrophages and dendritic cells to bypass CD4 T cell activation (62), MAIT cells may play an important role in circumventing these immune evasion mechanisms by *M. tuberculosis*.

In conclusion, our study suggests that frequencies of mycobacteria-reactive MAIT cells in the peripheral blood are not substantially modulated by BCG vaccination but are, rather, highly responsive to proinflammatory innate cytokines. Hence, MAIT cells are more likely to be adjuvants for other Ag-specific CD4 and CD8 T cells rather than to possess vaccine-induced memory properties themselves. This indirect role and its implication on TB pathogenesis should be explored in future studies of other candidate TB vaccines, particularly when administered through aerosol routes.

## Supplementary Material

Data Supplement
