# Supplementary material for: MR1-Independent Activation of Human Mucosal-Associated Invariant T Cells by Mycobacteria
Source: J Immunol. 2019 Oct 14;203(11):2917–27. doi: 10.4049/jimmunol.1900674 (PMC6859375; doi:10.4049/jimmunol.1900674)
Supplement: Data Supplement [file JI_1900674.zip › JI_1900674_Supplemental_Material_1.pdf]

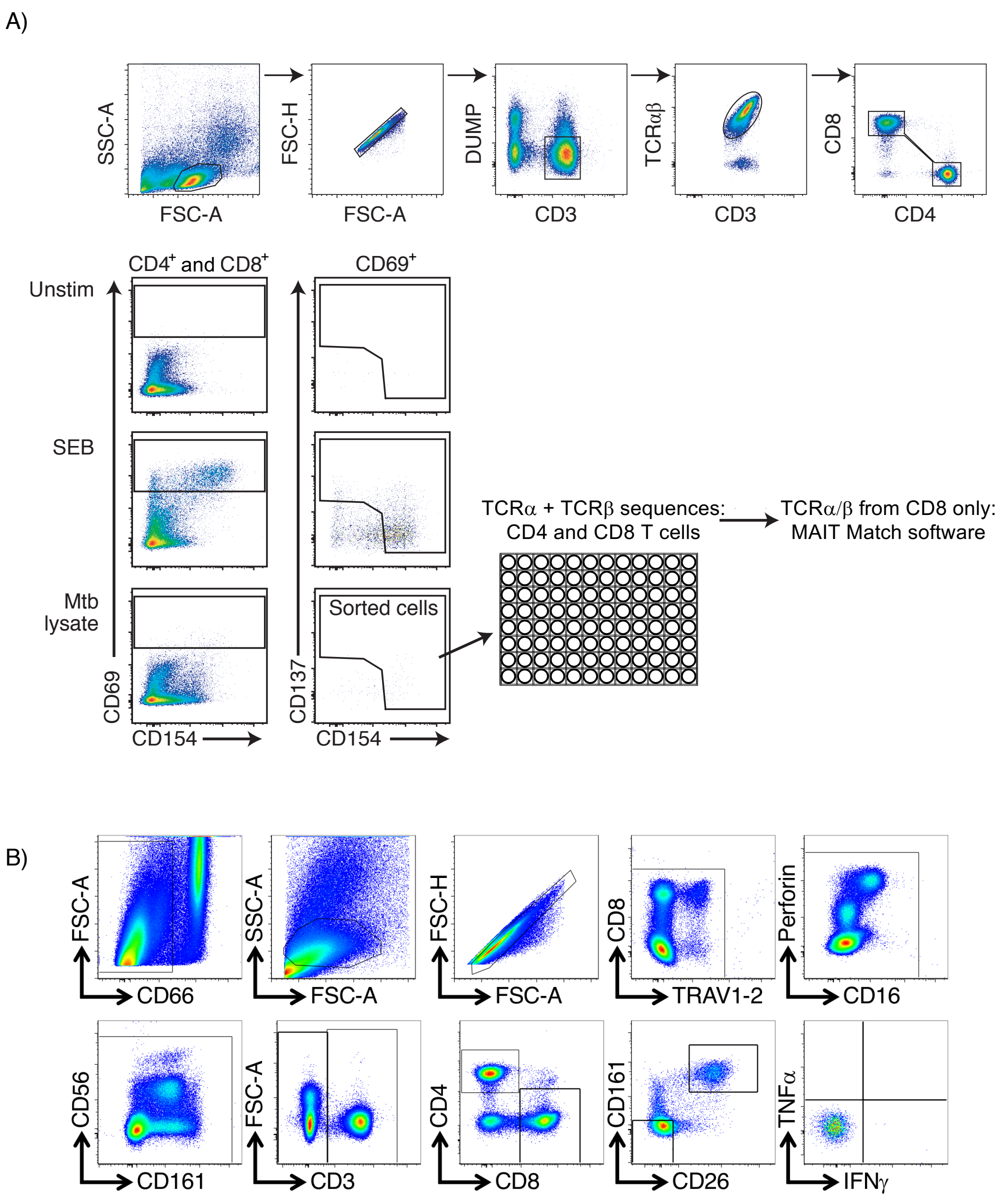

Supplementary Figure 1: Gating strategies for flow cytometry analysis

(A) Flow cytometry gating strategy and analysis pipeline to generate TCRα and TCRβ sequences from mycobacteria-reactive CD8 T cells following stimulation of PBMC samples with mycobacterial antigens. Unstim: unstimulated (negative control, top), SEB: Staphylococcus aureus Enterotoxin B super-antigen (positive control, middle), and *M.tb* lysate stimulation (antigen of interest, bottom). $CD69^{+}CD137^{+}$ or $CD69^{+}CD154^{+}$ "activated" T cells, including both CD4 and CD8 subsets, were sorted into 96 well plates and TCRα and TCRβ sequences were amplified from single cell cDNA templates. Sequences corresponding to wells with index-sorted $CD8^{+}$ T cells only were analyzed by the MAIT Match software.

(B) Revised gating strategy for flow cytometry analysis of whole blood intracellular cytokine staining assay after inclusion of anti-CD4 and anti-TRAV1-2 antibodies.

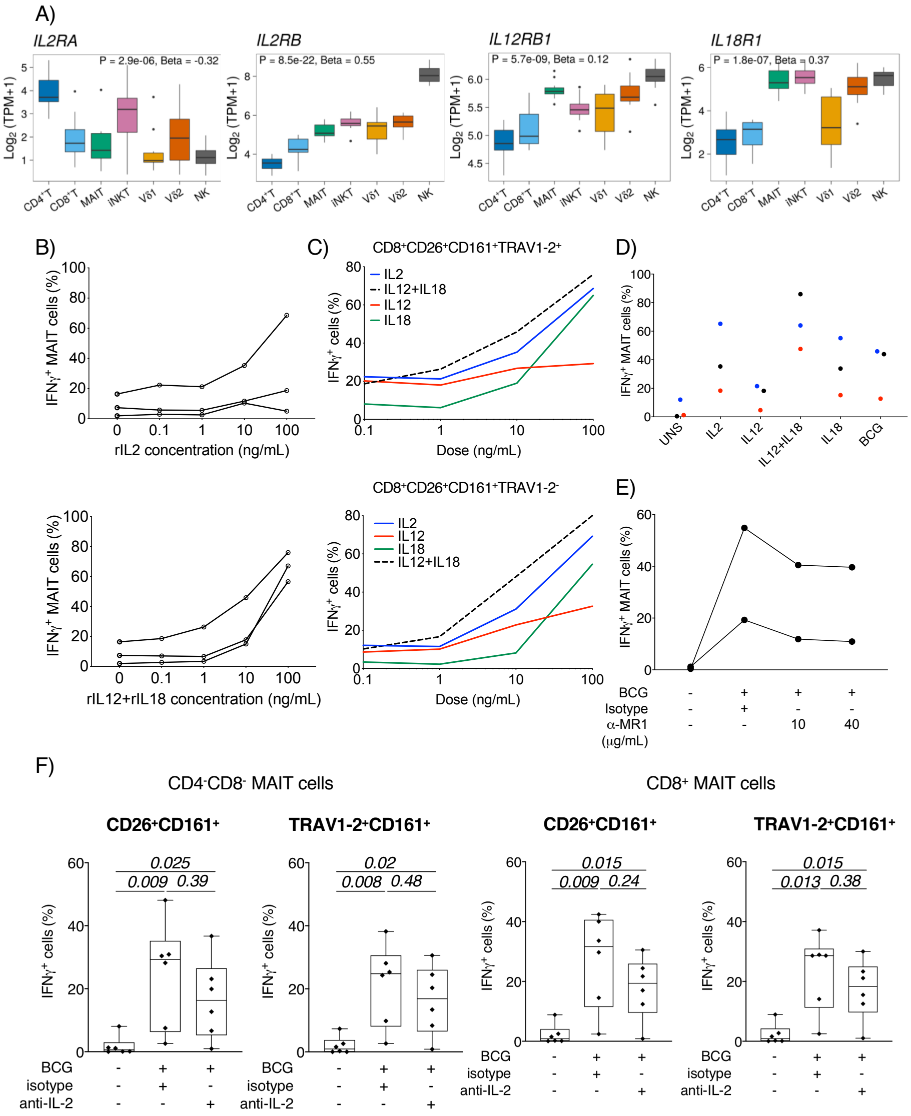

Supplementary Figure 2:

(A) Reported transcript levels of cytokine receptor genes: CD25 (IL-2Rα), CD122 (IL2Rβ), CD212 (IL2Rβ1), CD218a (IL18R1) from published low-input RNA-sequencing dataset (Gutierrez-Arcelus, et al., accession number: GSE81772) of adaptive (CD4 and CD8), and innate T cell populations (MAIT, iNKT and two γδ T cell subsets: Vδ1 and Vδ2), as well as natural killer (NK) cells, sorted from PBMC samples of healthy adult volunteers, using the online application in https://immunogenomics.io/itc/. The Beta and p-value measure whether the expressed gene is associated with the reported innateness gradient (Gutierrez-Arcelus, et al).

(B) Frequencies of IFNγ-expressing $CD8^+CD26^+CD161^+$ phenotypic MAIT cells in whole blood following stimulation with different doses of recombinant IL-2 (top) or a combination of recombinant IL-12 and IL-18 (bottom) from healthy individuals (n=3).

(C) Frequencies of IFNγ-expressing TRAV1-2-positive (top) or TRAV1-2-negative (bottom) $CD8^+CD26^+CD161^+$ phenotypic MAIT cells in whole blood following stimulation with different doses of recombinant IL-2 (blue), IL-12 alone (red), IL-18 alone (green), or a combination of IL-12 and IL-18 (dashed black) in a single donor.

(D) Frequencies of IFNγ-expressing $CD8^+CD26^+CD161^+$ phenotypic MAIT cells in response to different cytokines at 100ng/mL compared to BCG in whole blood samples from 3 different donors, each depicted by a different color.

(E) Frequencies of IFNγ-expressing $CD8^+CD26^+CD161^+$ phenotypic MAIT cells in unstimulated whole blood samples or following stimulation with BCG in the presence of a mouse IgG2a isotype control at 10μg/mL or anti-MR1 antibody at 10μg/mL or 40μg/mL. Lines connect paired whole blood samples from each of two healthy donors.

(F) Frequencies of IFNγ-expressing MAIT cells in unstimulated whole blood samples or following stimulation with BCG in the presence of isotype control or anti-IL-2 antibody. Box and whiskers depict median and interquartile ranges, respectively. *P*-values are calculated using the Wilcoxon t-test.

Supplementary Table1: Antibody panels used for flow cytometry analysis

Sample type	Fluorochrome	Antigen	Clone	Catalogue no.	Supplier
Adult whole blood samples for intracellular cytokine staining: BCG re-vaccination trial (Figures 1A, 2A-2D, 3A-3B) and healthy adults (Figures 5-7,and supplementary Figure 2)	APCH7	CD3	SK7	560176	BD
	PE (Figs. 1 & 2)	CD158b	CH-L	561723	BD
	PE (Figs. 3 & 4)	CD4	RPA-T4	555347	BD
	Brilliant-Violet-650	CD8	RPA-T8	563821	BD
	Brilliant-Violet-510	CD16	3G8	302048	Biolegend
	APC	CD26	BA5b	302710	Biolegend
	Brilliant-Violet-711	CD56	HCD56	318335	Biolegend
	FITC (or BB515)	CD66	B1.1/CD66	551479	BD
	PE-Cy5	CD161	DX12	551138	BD
	Brilliant-Violet-605 (Figs 1-3)	CD57	NK-1	563895	BD
	Brilliant-Violet-605 (Figs 5-7)	TRAV1-2	3C10	351720	Biolegend
	AlexaFluor700	IFNγ	B27	557995	BD
	Brilliant-Violet 421	Perforin	B-D48	353307	Biolegend
	PE-Cy7	TNFα	MAb11	25-7349-82	eBioScience (Thermofisher)
Infant whole blood samples for intracellular cytokine staining (delayed BCG vaccination cohort): Figures 3E and 3F	APCH7	CD3	SK7	560176	BD
	PE (Figs. 3E)	CD158b	CH-L	561723	BD
	PE (Figs. 3F)	CD4	RPA-T4	555347	BD
	Brilliant-Violet-650	CD8	RPA-T8	563821	BD
	Brilliant-Violet-510	CD16	3G8	302048	Biolegend
	APC	CD26	BA5b	302710	Biolegend
	Brilliant-Violet-711	CD56	HCD56	318335	Biolegend
	FITC (or BB515)	CD66	B1.1/CD66	551479	BD
	PE-Cy5	CD161	DX12	551138	BD
	Brilliant-Violet-605 (Figs 3E)	CD57	NK-1	563895	BD
	Brilliant-Violet-605 (Figs 3F)	TRAV1-2	3C10	351720	Biolegend
	AlexaFluor700	IFNγ	B27	557995	BD
	Brilliant-Violet 421	Perforin	B-D48	353307	Biolegend
	PE-Cy7	TNFα	MAb11	25-7349-82	eBioScience (Thermofisher)
Adult PBMC samples for surface staining to validate frequencies of CD26+CD161+ and MR1-tetramer-positive CD8 T cells (Figures 1B and 1C)	AF700	CD3	UCHT1	300424	Biolegend
	BV510	CD4	RPA-T4	300546	Biolegend
	BV785	CD8	SK1	344740	Biolegend
	BV605	CD26	M-A261	744450	BD
	PE Cy5	CD161	DX12	551138	BD
	PE Cy7	TRAV1-2	3C10	351712	Biolegend
	PE	MR1-tetramer (5-OP-RU loaded)	-	-	NIH tetramer core facility
	AF488	MR1-control tetramer (6-FP loaded)	-	-	NIH tetramer core facility
	NIR	Live/Dead	-	L10119	Thermofisher
	APC-H7	CD14	MφP9	560180	BD
	APC-H7	CD19	SJ25C1	641395	BD
Adult PBMC samples for sorting single cells for TCR sequencing analysis (Supplementary Figure 1 and Figure 4)	BV510	LIVE/DEAD (Aqua)	-	L34966	Life Technologies
	BV510	CD19	HIB19	302242	Biolegend
	BV510	CD14	M5E2	301842	Biolegend
	BV786	CD3	SK7	563800	BD
	APC	CD69	L78	340560	BD
	PE	CD154	TRAP1	555700	BD
	BV605	CD4	RPA-T4	300556	Biolegend
	AlexaFluor700	CD8	RPA-T8	561453	BD
	FITC	CD26	BA5b	302704	Biolegend
	BV421	HLA-DR	L243	307636	Biolegend
	PE-Cy7	TCRαβ	IP26	306720	Biolegend
	BV711	CD137	4B4-1	740798	BD
